# Assembly mechanisms of dung beetles in temperate forests and grazing pastures

**DOI:** 10.1038/s41598-019-57278-x

**Published:** 2020-01-15

**Authors:** Ilse J. Ortega-Martínez, Claudia E. Moreno, Cecilia Lucero Rios-Díaz, Lucrecia Arellano, Fernando Rosas, Ignacio Castellanos

**Affiliations:** 1 Centro de Investigaciones Biológicas, Instituto de Ciencias Básicas e Ingeniería, Universidad Autónoma del Estado de Hidalgo, Mineral de la Reforma, Hidalgo, Mexico; 2 Red de Ecoetología, Instituto de Ecología, A. C., Xalapa, Veracruz, Mexico

**Keywords:** Community ecology, Ecology, Biodiversity

## Abstract

The role of deterministic and stochastic mechanisms in community assembly is a key question in ecology, but little is known about their relative contribution in dung beetle assemblages. Moreover, in human modified landscapes these mechanisms are crucial to understand how biodiversity can be maintained in productive agroecosystems. We explored the assembly mechanisms driving dung beetle assemblages in forests and grazed grassland patches, and assessed the role of dung availability, soil hardness and moisture, elevation and land use heterogeneity as environmental predictors of functional diversity. To determine the underlying assembly mechanisms, we estimated functional diversity metrics (functional richness, evenness and divergence) and their departure from the predicted values by null models. We also used GLMs to assess the influence of environmental variables on functional diversity. In most cases, stochastic processes prevailed in structuring dung beetle assemblages and, consequently, environmental variables were not good predictors of dung beetle functional diversity. However, limiting similarity was found as a secondary mechanism with an effect on dung beetle assemblages in grasslands. Our results highlight the importance of stochastic processes that may reflect a metacommunity dynamic. Therefore, restoring landscape connectivity might be more important than habitat quality for the conservation of these functionally diverse beetle assemblages.

## Introduction

Community assembly theory provides a conceptual foundation about the mechanisms that determine species composition of local assemblages^1,2^, and this background becomes especially relevant for studies concerning the ecological consequences of environmental contemporary changes. For example, results from a recent study have made it possible to improve our understanding of biological invasions, a global issue that is critical in ecology and conservation^3^. However, an important remaining issue is to unravel how current anthropogenic changes to the environment, such as land use change and agricultural production can modify the assembly mechanisms that shape the structure and composition of local communities^4^. Human activities have contributed substantially to the loss of species and degradation of ecosystems^5,6^, generating impacts on ecological communities^7^. Therefore, identifying the drivers of community assembly in human modified landscapes is an urgent challenge.

Both deterministic and stochastic mechanisms are important in assembling biological communities. The main deterministic mechanisms driving community assembly are limiting similarity and environmental filtering^8–10^. If the local community is formed by non-redundant species, its trait diversity will be high (trait overdispersion) as a result of strong ecological interactions, and community assembly is considered to be driven by a limiting similarity mechanism due to competition^8^. On the contrary, the environmental filtering mechanism (the abiotic filter) structures local communities through environmental restrictions^10^ producing sets of species with similar traits (trait clustering)^8,11,12^. Besides environmental filtering *sensu stricto*, recent advances in coexistence theory have shown that there are other mechanisms that may led to trait clustering^10^, such as competitive ability differences (average fitness differences)^13^, mutualism^14^ or facilitation^10^. Therefore, if trait clustering is found in empirical studies, a clear framework should be followed to discern between environmental filtering *sensu stricto* and ecological interaction mechanisms^10^. In a different way, the stochastic assembly of communities relies on the dispersal of individuals across space, regardless of their traits, so the structure and composition of communities is limited by ecological drift and the species’ dispersal capacity, a process related to the neutral theory^15^.

Several environmental conditions may change the relative influence of limiting similarity, environmental filtering and stochastic mechanisms on community assembly, particularly if those conditions alter dispersal, arrival, and niche availability. Therefore, our ability to understand the relative importance of these not mutually exclusive assembly mechanisms is critical, as they can have high impacts on the conservation of local biodiversity and ecological functions. For example, a poor knowledge about the drivers of community assembly limit our capacity to predict the consequences of land-use change on diversity, and to devise useful management interventions in man-made landscapes^4^.

An effective quantitative approach for understanding community assembly mechanisms is the use of functional trait-based diversity metrics^8,9,16,17^. For example, recent studies on ground-dwelling beetles have incorporated the analysis of functional traits to assess forest-grassland gradients^18^ and community assembly rules^19^. Because they are related to species’ niches, the variability of those traits within communities (i.e., functional diversity) is assumed to reflect the imprint of assembly mechanisms such as environmental filtering or competitive interactions. To disentangle the relative contributions of assembly mechanisms, we can measure the deviation of the observed functional diversity estimates from their expected distribution under a random model of assembly. Such differences are then interpreted in the light of theoretical frameworks and the most likely assembly mechanism is inferred (e.g., Liu & Wang^20^). If the functional diversity of coexisting species is higher than expected by chance, we would infer a mechanism of limiting similarity that produces over-dispersion in functional traits. On the contrary, if the functional diversity of coexisting species is lower than expected by chance, we would expect an environmental filtering mechanism, producing under-dispersion in functional traits (i.e., functional convergence). Finally, if the observed functional diversity is not different from the expected in a null model of random assembly, stochastic processes are assumed.

Dung beetles (Coleoptera: Aphodiinae, Geotrupinae and Scarabaeinae) use vertebrate dung (mainly that of large and medium sized mammals) for food and nesting^21^. They are an ideal group for studying the impact of human activities on community assembly due to their responses to land use change, as well as to their functional trait diversity. They perform several ecosystem functions, including dung decomposition through their burial and removal, and nutrient recycling. These primary functions have consequences on secondary functions such as bioturbation, secondary seed dispersal and greenhouse gases control^22–25^. Studies on dung beetle functional diversity have shown changes across different land uses and livestock production systems^26,27^. Moreover, a recent study based on the functional diversity of dung beetles suggests that the environment restricts species dispersion or establishment along an elevation gradient, thus a niche filtering mechanism would be structuring these communities^28^. Likewise, Audino *et al*.^29^ found that niche-based processes drove the assembly of dung beetle assemblages following active restoration, mainly by the influence of environmental filters. However, it is unlikely that a single process would be responsible of the assemblages’ structure. Hence, to understand the assembly mechanisms of dung beetle assemblages in landscapes with human impacts, it would be relevant to assess the combined effects of deterministic and stochastic mechanisms. By estimating functional diversity metrics and their departure from expected values of null distributions, we evaluate assembly mechanisms structuring dung beetle assemblages in forests and grassland patches where livestock graze in a mountain landscape. Additionally, we assess the influence of environmental variables (dung availability, soil hardness and moisture, elevation and land use heterogeneity) over functional diversity metrics. Due to the contrasting environmental conditions between forest and grasslands, and the availability of food resources that reduces competition, we expect that locally coexisting species will have similar traits due to an environmental filtering mechanism. According to this, functional diversity will be explained by environmental conditions.

## Results

We recorded 3,639 individuals belonging to 23 species of subfamilies Aphodiinae, Geotrupinae and Scarabaeinae. Overall, dung beetles were more abundant and diverse in grazed grasslands (2,590 individuals of 19 species) than in forest sites (1,049 individuals of 14 species). The most abundant species in both conditions were *Onthophagus mexicanus* Bates, 1887 (n = 1,544 individuals), *Gonaphodiellus opisthius* (Bates, 1887) (n = 1,057), *Onthophagus chevrolati* Harold, 1869 (n = 341), *Phanaeus palliatus* Sturm, 1843 (n = 139) and *Copris armatus* Harold, 1869 (n = 139). From the total number of individuals, 64% are tunnellers, 34% are dwellers, while only 2% are rollers (Supplementary Table S1).

### Assembly mechanisms

**Figure 1:**
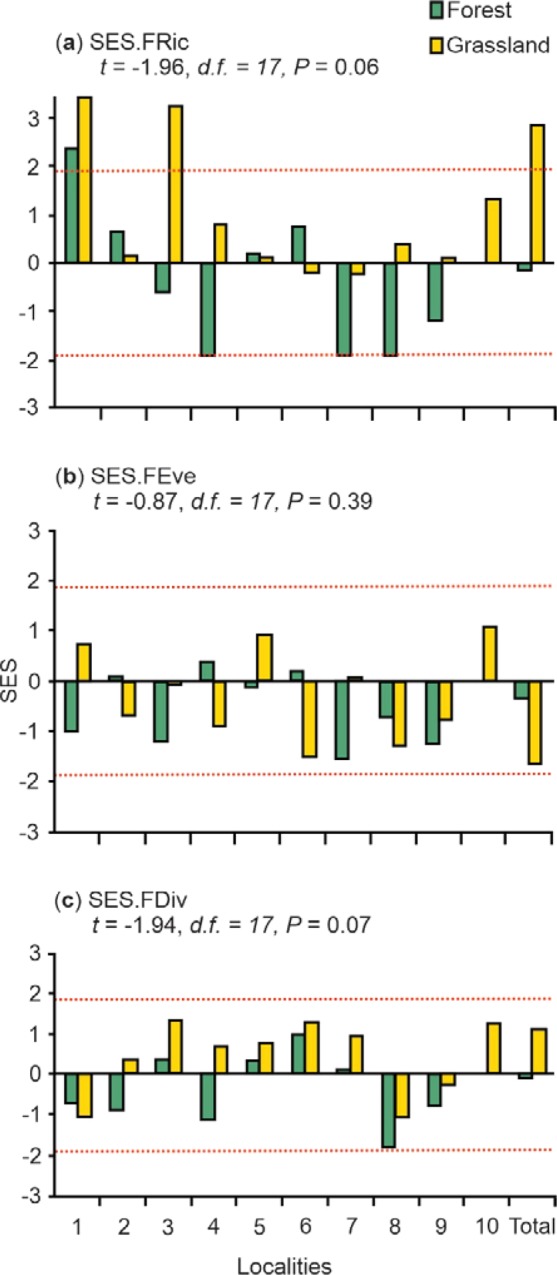
Standardized effect size (SES) of functional richness (**a**), functional evenness (**b**), and functional divergence (**c**) of dung beetle assemblages in forest and grassland sites, and the total cumulative data of each environmental condition. The localities have been ordered from lowest (to the left) to highest (to the right) elevation (Supplementary Table S2). The red dotted lines include the values that are not statistically different from a distribution expected by chance, according to null models (−1.96 to 1.96 interval). Values of t tests comparing forest and grassland sites are included.

The SES values for the three FD metrics used (FRic, FEve and FDiv) were not statistically different between forest and grassland (P > 0.05, Fig. 1). Most of the sites had SES values within the randomly expected null interval, thus stochastic processes are assumed to be the main forces structuring these dung beetle assemblages. Nevertheless, SES.FRic values from one forest and two grassland sites (low elevation), were greater than 1.96, indicating limiting similarity.

When we pooled the data from all sampling sites of each environmental condition, the SES values of the three FD metrics were negative for the pine-oak forest, but not lower than the randomly expected values. However, for grassland the SES.FRic value was higher than expected by chance, indicating that limiting similarity shapes dung beetle assemblages in these grazed grasslands (Fig. 1).

### Environmental predictors of functional diversity

For functional richness the best-fitted model included elevation and dung availability as predictor variables when all the sites were considered, but only elevation had a significant negative relationship (Table 1). However, when the forest and grassland sites were analyzed separately, any of the environmental variables had a significant relationship with functional richness. Similarly, none of the environmental predictors had a significant relationship with functional evenness.

**Table 1:** Generalized linear models (GLMs) for the standardized effect sizes of functional richness (SES.FRic), functional evenness (SES.FEve), and functional divergence (SES.FDiv) of dung beetle assemblages in response to environmental variables. We show with an asterisk the variables included in best-fitted models (lower AIC), and the sign for significant relationships; “–” indicates the variables not included in best-fitted models (see other models in Supplementary Table S3).

	All sites	Forest sites	Grassland sites
**Functional richness**			
Elevation	* (−)	*	*
Dung availability	*	*	—
Soil moisture	—	*	—
Soil hardness	—	*	—
Land use heterogeneity	—	*	—
Percentage of pine-oak forest	—	*	—
Percentage of grassland	—	*	—
Null Deviance	42.93	17.96	17.01
Residual Deviance	24.46	0.10	11.05
AIC	66.72	2.71	35.38
**Functional evenness**			
Elevation	—	—	—
Dung availability	—	—	—
Soil moisture	—	—	—
Soil hardness	—	—	—
Land use heterogeneity	*	—	—
Percentage of pine-oak forest	—	*	—
Percentage of grassland	—	—	*
Null Deviance	12.17	4.08	7.56
Residual Deviance	11.25	2.85	5.38
AIC	49.97	21.18	28.18
**Functional divergence**			
Elevation	–	*	*
Dung availability	–	*	* (-)
Soil moisture	* (+)	*	*
Soil hardness	—	*	—
Land use heterogeneity	—	—	—
Percentage of pine-oak forest	—	*	—
Percentage of grassland	—	*	—
Null Deviance	16.52	6.20	7.31
Residual Deviance	12.29	0.89	0.80
AIC	51.64	20.67	13.10

Finally, the best-fitted model showed that functional divergence values increased with higher soil moisture when we considered all the sites, while for grassland sites we detected a negative relationship between functional divergence and dung availability (Table 1).

## Discussion

Contrary to our expectation, stochastic processes prevail in structuring the studied dung beetle assemblages. Accordingly, environmental variables were not good predictors of dung beetle functional diversity. These results reflect some degree of ecological equivalence among coexisting dung beetle species, given the lack of clear trends of functional divergence (over-dispersion) or convergence (under-dispersion)^30^. Therefore, the dynamics of dung beetle assemblages may depend on processes related to species’ dispersion and demographic stochasticity^31,32^. A similar pattern has also been detected for ground beetles (Carabidae) in urban and suburban areas, where communities are randomly assembled as a result of stochastic mechanisms^33^.

From the point of view of the spatial dynamics of community ecology, our samples of beetles in both habitats are part of a regional metacommunity, thus the set of local assemblages may be connected by dispersion^34^. In this scenario, despite their contrasting differences in tree coverage, food availability and environmental conditions, the grassland patches could be so small that may represent a continuous environment with the forest matrix, allowing the existence of a neutral dynamic for dispersal^35^. Such a neutral dynamic assumes that, given random changes in their abundance, species have the same competitive capacity in both habitats, thus their coexistence depends on the ecological drift (an equilibrium between immigration and extinction processes^15^). A similar pattern has been recently described for ground beetles in grasslands embedded within a forest matrix in the northern Hungarian mountains, where a random assembly structure is explained by the considerable asymmetrical species flow (spillover) from adjacent forests^19^. Although in our study area dung beetle species richness, abundance and diversity are higher in grasslands than in forest interior, 41% of the species have been caught in both habitats (Rios-Díaz *et al*., unpublished data). This indicates that there is a significant flow of individuals and species between habitats probably because forests can be used by some species as habitat, and they move to the adjacent grasslands for feeding. Therefore, in order to test the metacommunity hypothesis, we would require detailed information on population’s movements.

Dispersal capacity varies among dung beetle species. Some of them can move 50 to 100 m in few days^36,37^ or even up to 1.5 km, although this depends on their physiological (reproductive) condition or sex^38,39^. In open environments some generalist species show high vagility, while specialist species are less vagile^40^. Also, large species move more frequently in grasslands than small species^41^. However, as far as we know, the spatial movements of dung beetles have not been assessed in temperate mountainous landscapes similar to our study area, therefore capture-recapture experiments or remote sensing devices will be required to describe their movement patterns.

Besides their biological restrictions, landscape elements also have differential impacts on the dispersal capacity of species. For example, open areas may act as barriers for the dispersion of some forest specialist species^28,38,42–44^, because temperature and moisture conditions change^45^. However, some forest patches and other landscape elements such as live fences and isolated trees promote spatial heterogeneity and can increase functional connectivity, reducing the effects of isolation^38,46^. In our study area, grassland patches include several *Agave* fences that may act as corridors for insects. Also, the dispersal dynamics of dung beetles, as well as their opportunity to get food, may be related to the movement and density of livestock^47^.

Again, contrary to our expectation, we found that one forest, two grassland sites, and the complete set of grassland species, showed functional over-dispersion, which is assumed to be a result of limiting similarity processes. This suggests that, despite the availability of food resources (mammal dung), coexisting dung beetle species are functionally different in some grasslands. This could represent a secondary factor that contributes simultaneously with the stochastic process to the observed functional structure. Ecological theory would expect ecological competition to be a result of low resource availability^48–50^, and strong competitive interactions are expected to produce functional divergence in coexisting species^8^. In temperate forests of Northern Europe, competitive interactions influence the structure of dung beetle assemblages due to the limited availability and size of dung^47^. However, this does not seem to be the case in our study, as we even found a negative relationship between dung availability and functional divergence in grassland sites. Therefore, in the grasslands of our study region, we still need to assess other characteristics of dung, beyond its frequency on the ground. For example, traditional livestock movement may result in a complex spatial mosaic of dung with different degrees of quality and freshness. Moreover, the composition of dung beetle assemblages in grasslands may be also influenced by the intensity and history of grazing^51,52^.

It has been proposed that dung beetle assemblages in tropical forests are structured by their niche space (environmental filtering), while the influence of limited dispersal and random colonization are discarded^29^. This suggests that the arrival and establishment of species depends on environmental factors^29,53^, and could result in ecological redundancy in dung beetle assemblages^28,54,55^. Also, in tropical forest the degradation and loss of natural habitats decreases the functional diversity of dung beetles^26,27,56^, which has consequences on their ecosystem functions^57^. However, in our study the lack of difference in functional diversity metrics between forest and grassland sites suggest that functional diversity of dung beetle assemblages is not affected by grazing in grassland patches. A similar situation has been found in scrub habitats near our study area^26^, and in the Cerrado biome in Brazil, where functional diversity does not change among vegetation types^58^.

In summary, our results indicate that in a temperate forest landscape, dung beetle assemblages are mainly determined by stochastic processes, and to a lesser extent by the deterministic process of limiting similarity. Therefore, these processes are not mutually exclusive, and may be acting jointly in structuring beetle communities under metacommunity dynamics associated to the traditional pastoral activities in the small grasslands of this montane landscape.

Future research will be required to know if these dynamics vary with increasing land use change or more intensive livestock management. In such cases, dung beetle assemblages could be facing the opposite processes: ecological disassembly mechanisms due to species loss. Therefore, better knowledge on assembly mechanisms might be useful to develop appropriate restoration policies. For example, Wearn *et al*.^4^ suggest that if environmental filtering is the dominant driver of assembly, then steps to restore habitat quality may be important. Alternatively, if dispersal limitation dominates assembly, then restoring landscape connectivity might be more important. However, determining specific conservation practices will require complementary information, such as data on alpha and beta taxonomic and phylogenetic diversity, and temporal dynamics of dung beetle species.

## Materials and Methods

### Study area

The study was carried out at the UNESCO Global Geopark Comarca Minera^59^ located in the state of Hidalgo, central Mexico, in the Mexican Transition Zone (Supplementary Method: Study area and location of sampling sites; Supplementary Fig. S1). In this mountainous area, livestock production is based on small flocks of sheep and goats that are guided by a shepherd in open grassland patches embedded in a forest matrix. Vegetation is characterized by pine-oak forest where *Pinus teocote, P. montezumae*, *P. patula*, *Quercus laurina*, *Q. crassifolia* and *Q. rugosa* are the most frequent tree species.

### Data collection

Ten localities separated each other by at least 1 km were selected at the study region. At each locality we set two sampling sites: one in a grazed grassland and the other in the contiguous pine-oak forest. The minimum distance between the two sampling sites within each locality was 300 m, and each site was at least 100 m from the forest edge in order to avoid edge effects, as they may have strong influence on beetle species composition^60,61^. Sites ranged from 2,200 to 2,726 m a.s.l. (Supplementary Table S2).

As our research question is not related to temporal variation in the studied assemblages, we did not take into account seasonal or annual dynamics. Instead, dung beetles were sampled during the rainy season (August and September 2016), when they are most active, to maximize capture success. Previous studies in our study area have proven that intensive sampling during this limited period may be enough for recording dung beetle assemblages properly^62^. Moreover, we assessed the completeness of our samples to represent dung beetle assemblages at each sampling site (see below).

We used baited pitfall traps made of a plastic container (1 litre) buried at soil level, with ethylene glycol diluted in 10% water (250 ml) to break the surface tension and to preserve the beetles. Each pitfall trap was baited with a mixture (3:1) of sheep and horse dung (ca. 250 g). All this sampling methodology has been successfully used in the region for sampling dung beetles, as big native mammals are extremely scarce, thus, livestock is the main source of dung^62^. At each sampling site, we set up nine pitfall traps separated 50 m (following Larsen & Forsyth^36^). Although other authors have suggested larger distances for pitfall trapping, we set this distance as we are not using traps as replicates, but as subsamples in order to complete the species set of each site. Traps were left open 144 h and were baited with fresh dung every 48 h. Beetles were identified to the species level using a local reference collection of Scarabaeidae, following taxonomic keys^63–71^ and with the help of specialists (Fernando Escobar and Pablo Minor Montiel from the Instituto de Ecología, A.C., Xalapa, México).

### Trait data

To calculate functional diversity (FD) metrics, we selected behavioural, dietary and morphological traits of species, as these have been proposed as key characteristics in the response of terrestrial invertebrates to environmental change, and that may affect ecosystem processes^72^. The traits considered in this paper are: 1) food relocation behaviour, 2) activity period, 3) diet type, 4) morphological measurements (body length, body width, dorso-ventral length, clypeus length, head length, pronotum length, abdomen length, forelegs length and hindlegs length), 5) wing loading, and 6) biomass (see details in Supplementary Method: Functional traits of dung beetle species; Supplementary Table S4).

### Community parameters

As a preliminary analysis, we calculated the sample coverage^73^ to assess the completeness of our samples to represent dung beetle assemblages at each sampling site. In general, the estimates of sampling coverage were higher than 0.91 in all but in one forest site (0.85), indicating that the sampling effort was enough to achieve a good representation of assemblages. However, for the following analyses one forest site was omitted because of its low richness and abundance (Supplementary Table S1).

Although there are several indices of functional diversity, we selected three (FRic, FEve, FDiv) proposed by Villéger *et al*.^74^ that have been suggested as the most appropriate metrics to assess assembly mechanisms^9^. Functional richness (FRic) represents the functional space (volume in the N dimensional space) that is occupied by the species in the community^74,75^. Functional evenness (FEve) measures the uniformity of the distribution of species and their abundance in the functional space, while functional divergence (FDiv) represents how abundance is distributed within the volume of functional trait space occupied by species^74–76^. We calculated FRic, FEve and FDiv for each sampling site and for the cumulative data of each environmental condition. These indices are based on the functional space defined by the species’ traits. To achieve this, a Principal Coordinates Analysis (PCoA) is produced to discard potentially correlated traits and reduce dimensionality. The resulting PCoA axes are used as the new standardized traits to compute the functional diversity indices, avoiding redundancy and biases. All the analyses were done using the dbFD function in the FD package^77,78^ in R 3.5.1^79^.

### Null models

Null models are commonly used to prove if observed patterns in ecological communities are significantly different from values expected by chance^30,80–83^. Therefore, we calculated expected values of FRic, FEve and FDiv indices by constructing null models to detect the relative contribution of different assembly mechanisms in structuring dung beetle assemblages^9^.

To generate null models, we shuffled the names of species on the trait data matrix to produce random combinations from the species pool: the set of species present in the region due to biogeographical and historical processes^84–86^. Then, we constructed null models using FD^77,78^ and picante^87^ packages in R 3.5.1^79^, following Swenson^80^. We created 999 random assemblages by randomizing the trait data while maintaining the community. Randomizations were carried out for the whole set of forest and grassland sites, considering that they might have the same species regional pool. Then, we used the observed and null expected values to quantify the standardized effect size (SES) of each index^88^. This calculation removes any directional bias associated with the decrease in variance in the expected values with increasing species richness^80^. To calculate the SES, we subtracted the mean value of the null distribution (mean expected value) from the observed value of functional diversity, and then divided it by the standard deviation of the null distribution^80^.

SES values can be positive or negative, and their statistical significance at P < 0.05 is assumed when the SES value falls outside the range of −1.96 to 1.96, assuming a normal distribution of deviations^9,20^. On one hand, a SES value greater than 1.96 indicates that there is functional over-dispersion due to a limiting similarity mechanism. On the other hand, SES values lower than −1.96 indicate that there is functional under-dispersion, which means that an environmental filtering mechanism occurs. When SES values fall within the range of −1.96 to 1.96, mechanistic processes cannot be proved, and then stochastic processes are assumed as the observed values cannot be distinguished from random values. SES values were calculated for each sampling site, and for the cumulative data of each environmental condition (forest and grassland).

### Environmental variables

We measured soil hardness, soil moisture and dung availability, at each sampling site, as these variables are related with beetles’ dung removal^89,90^. To measure soil hardness and moisture we took nine values per site, near to each pitfall trap. Soil hardness was measured with a graduated penetrometer (scaled 0 to 5) and soil moisture with an analogic soil moisture meter (scaled 0 to 10). We measured dung availability in four 20 m long transects at each site. Along each transect we located 1 m^2^ plots separated 1 meter and counted the frequency of dung occurrence in the 40 plots. We also measured elevation and land use heterogeneity surrounding pitfall traps. For this latter, we used aerial images taken with a drone (Phantom 3 advanced model, 12-megapixel camera, GPS + GLONASS) and measured the proportion of each land use type in a 100 m buffer around the pitfall traps (see details in Supplementary Method: Method used to characterize the heterogeneity of land uses surrounding the sampling sites; Table S5). Then, we calculated the exponential of the Shannon-Wiener index to estimate the effective number of land uses as a proxy of land use heterogeneity at each site.

### Statistical analyses

First, we checked for spatial autocorrelation of each functional diversity metric using Moran I correlograms^91^, and none spatial autocorrelation was detected (Moran’s I = −0.03, −0.11, and 0.16 for SES.FRic, SES.FEve and SES.FDiv, respectively, P > 0.05 in all cases). Then, we performed t tests to assess differences in the mean SES values of each functional diversity metric between forest and grassland sites, as these variables passed normality tests (Shapiro-Wilk tests, SES.FRic: W = 0.92, P = 0.16; SES.FEve: W = 0.94, P = 0.29; SES.FDiv: W = 0.93, P = 0.18).

To assess the relationships between the SES values of FD metrics (response variables) and environmental variables (predictors) we used generalized linear models (GLMs). We used gaussian distributions as the response variables were normally distributed (see above). As predictors we included all possible combinations of the environmental variables (soil hardness, soil moisture, dung availability, elevation and land use heterogeneity). We first ran the models for all the sites, and then separately for each environmental condition (forest and grassland). The candidate models were compared using the Akaike information criterion (AIC)^92^. Models with the lowest AIC values were selected as the best models^93^. All analyses were done in R 3.5.1^79^.

## Supplementary information

Supplementary information.

## Data Availability

All data generated during this study are included in this published article (and its Supplementary Information files online).
