# Supplementary material for: Assembly mechanisms of dung beetles in temperate forests and grazing pastures
Source: Sci Rep. 2020 Jan 15;10:391. doi: 10.1038/s41598-019-57278-x (PMC6962461; doi:10.1038/s41598-019-57278-x)
Supplement: Supplementary file 1 — Supplementary information. [file 41598_2019_57278_MOESM1_ESM.docx]

**[Supplementary Information](https://www.nature.com/articles/s41598-019-49027-x#Sec16)**

**Assembly mechanisms of dung beetles in temperate forests and grazing pastures**

Ilse J. Ortega-Martínez^1^, Claudia E. Moreno^1*^, C. Lucero Rios-Díaz^1^, Lucrecia Arellano^2^, Fernando Rosas^1^ and Ignacio Castellanos^1^

^1^ Centro de Investigaciones Biológicas, Instituto de Ciencias Básicas e Ingeniería, Universidad Autónoma del Estado de Hidalgo, Mineral de la Reforma,

Hidalgo, Mexico

^2^ Red de Ecoetología, Instituto de Ecología, A. C., Xalapa Veracruz, Mexico

^*^Corresponding Author: Claudia E. Moreno

Email: [cmoreno@uaeh.edu.mx](mailto:cmoreno@uaeh.edu.mx)

**Table S1. Parameters of dung beetle assemblages at each forest and grassland site, and for cumulative data (total) of both conditions.**

| Forest | | | | | | | Grassland | | | | | |
| --- | --- | --- | --- | --- | --- | --- | --- | --- | --- | --- | --- | --- |
| Locality | Richness | Abundance | Cn | FRic | FEve | FDiv | Richness | Abundance | Cn | FRic | FEve | FDiv |
| 1 | 7 | 563 | 0.996 | 0.031 | 0.415 | 0.954 | 8 | 429 | 0.998 | 0.017 | 0.332 | 0.993 |
| 2 | 3 | 12 | 0.917 | 0.010 | 0.639 | 0.863 | 8 | 483 | 0.994 | 0.205 | 0.489 | 0.796 |
| 3 | 9 | 238 | 0.996 | 0.258 | 0.616 | 0.806 | 15 | 973 | 0.997 | 0.213 | 0.471 | 0.670 |
| 4 | 3 | 11 | 1 | 0.151 | 0.59 | 0.869 | 9 | 198 | 0.990 | 0.186 | 0.612 | 0.791 |
| 5 | 8 | 145 | 0.979 | 0.198 | 0.445 | 0.816 | 7 | 86 | 0.977 | 0.186 | 0.452 | 0.779 |
| 6 | 3 | 14 | 0.857 | 0.001 | 0.392 | 0.591 | 6 | 100 | 0.990 | 0.176 | 0.668 | 0.673 |
| 7 | 3 | 18 | 1 | 0.151 | 0.998 | 0.754 | 8 | 106 | 0.991 | 0.222 | 0.542 | 0.750 |
| 8 | 3 | 22 | 0.955 | 0.151 | 0.817 | 0.978 | 3 | 22 | 0.955 | 0.023 | 0.937 | 0.894 |
| 9 | 4 | 26 | 1 | 0.18 | 0.884 | 0.888 | 7 | 115 | 0.974 | 0.184 | 0.504 | 0.866 |
| 10 | -- | -- | -- | -- | -- | -- | 5 | 78 | 1 | 0.043 | 0.374 | 0.613 |
| Total | 14 | 1049 | 0.999 | 0.275 | 0.503 | 0.88 | 19 | 2590 | 1 | 0.260 | 0.619 | 0.752 |

Cn: sample coverage, FRic: functional richness, FEve: functional evenness, FDiv: functional divergence.

**Supplementary Method: Study area and location of sampling sites**

The study was carried out in the state of Hidalgo, central Mexico (Fig. S1). It includes two geological provinces: Trans-Mexican Volcanic Belt and Sierra Madre Oriental, with three soil types: luvisol, andosol and lithosol. Mean annual precipitation is 575.7 mm, with a mean minimum temperature of 13.2 ºC and a mean maximum of 15.8 ºC^1^. We selected ten localities, and two sampling sites at each locality: one in grassland and one in pine-oak forests (Table S2). Field sampling was carried out under the scientific sampling permit FAUT-0254 (SEMARNAT, Mexico).


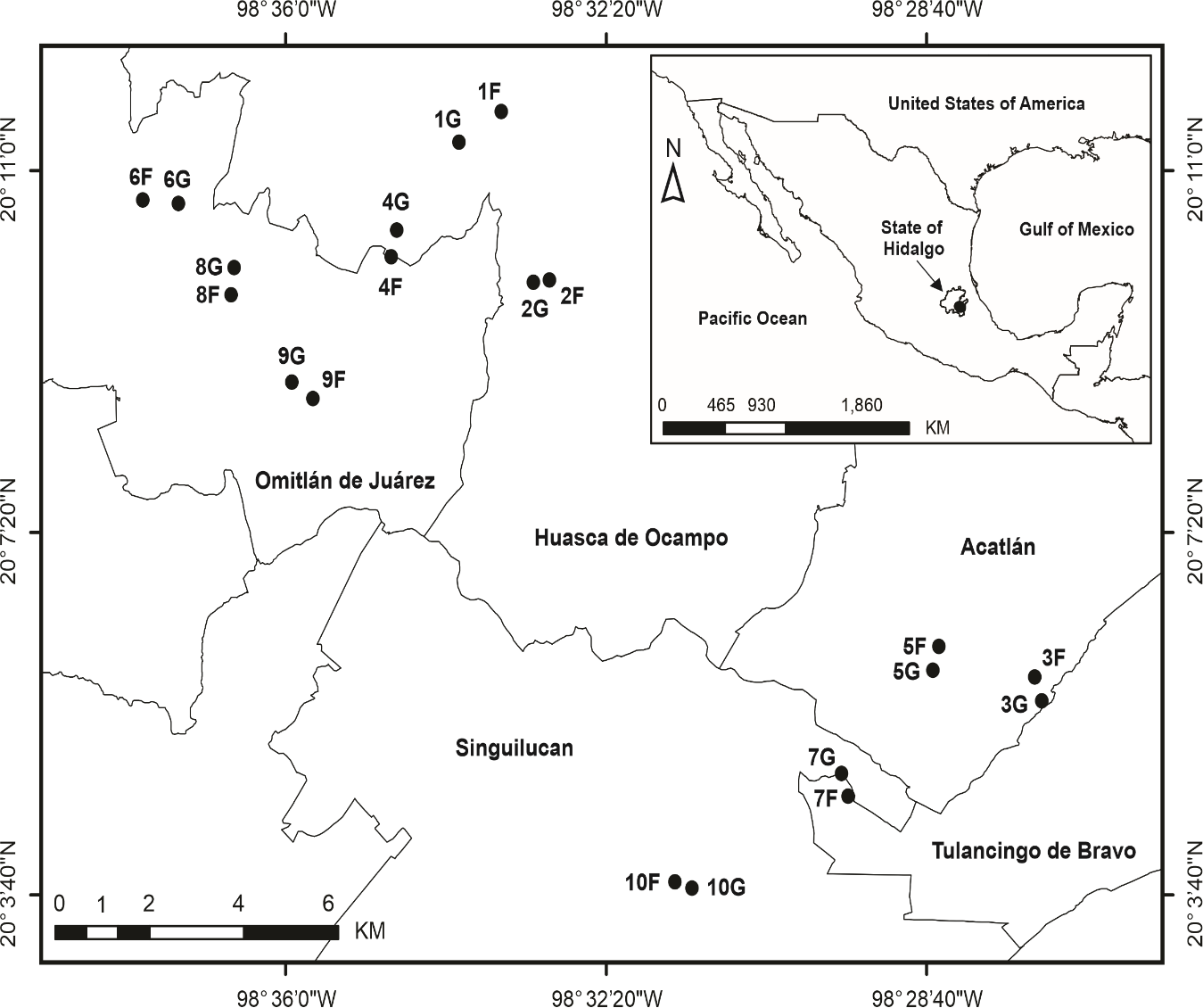
 **Figure S1**. Sampling sites for dung beetle assemblages in the Comarca Minera Geopark, state of Hidalgo, central Mexico. The lines in the lower figure denote municipality limits. F = forest sites, G = grassland sites. This map was created by Ilse Jaqueline Ortega-Martínez using Corel Draw Graphics Suite X4 (https://www.coreldraw.com/la/product/coreldraw/), based on the Digital Map of Mexico, which is freely available from the Instituto Nacional de Estadística y Geografía (INEGI, http://gaia.inegi.org.mx/).

**Table S2. Location of the 20 sampling sites in Hidalgo, Mexico. F = forest sites, G = grassland sites.**

| **Locality** | **Municipality** | **Site** | **Elevation** | **Latitude** | **Longitude** |
| --- | --- | --- | --- | --- | --- |
| 1. Llano Grande | Huasca de Ocampo | 1F | 2200 | 20° 11’ 35.53’’ | 98° 33’ 31.75’’ |
|  |  | 1G | 2215 | 20° 11’ 16.87’’ | 98° 34’ 0.76’’ |
| 2. Truchas El Zembo | Huasca de Ocampo | 2F | 2264 | 20° 9’ 53.32’’ | 98° 32’ 58.79’’ |
|  |  | 2G | 2216 | 20° 9’ 51.89’’ | 98° 33’ 9.79’’ |
| 3. Huajomulco | Tulancingo de Bravo | 3F | 2276 | 20° 5’ 52.22’’ | 98° 27’ 25.64’’ |
|  |  | 3G | 2246 | 20° 5’ 37.72’’ | 98° 27’ 20.75’’ |
| 4. Ejido Ixtula-Sembo | Huasca de Ocampo | 4F | 2430 | 20° 10’ 7.34’’ | 98° 34’ 47.44’’ |
|  |  | 4G | 2383 | 20° 10’ 23.36’’ | 98° 34’ 43.40’’ |
| 5. Ejido Acatlán | Acatlán | 5F | 2464 | 20° 6’ 10.75’’ | 98° 28’ 31.29’’ |
|  |  | 5G | 2484 | 20° 5’ 56.14’’ | 98° 28’ 35.45’’ |
| 6. Manuel Teniente | Omitlán de Juárez | 6F | 2582 | 20° 10’ 41.77’’ | 98° 37’ 37.84’’ |
|  |  | 6G | 2549 | 20° 10’ 39.70’’ | 98° 37’ 13.20’’ |
| 7. La Lagunita | Singuilucan | 7F | 2569 | 20° 4’ 39.86’’ | 98° 29’ 33.74’’ |
|  |  | 7G | 2576 | 20° 4’ 53.41’’ | 98° 29’ 38.20’’ |
| 8. Cerro Gordo | Omitlán de Juárez | 8F | 2635 | 20° 9’ 44.35’’ | 98° 36’ 37.25’’ |
|  |  | 8G | 2617 | 20° 10’ 0.73’’ | 98° 36’ 35.28’’ |
| 9. Mixquiapan | Omitlán de Juárez | 9F | 2679 | 20° 8’ 41.06’’ | 98° 35’ 40.96’’ |
|  |  | 9G | 2683 | 20° 8’ 51.25’’ | 98° 35’ 55.55’’ |
| 10. Rincón del Puerto | Singuilucan | 10F | 2726 | 20° 3’ 47.6’’ | 98° 31’ 32.66’’ |
|  |  | 10G | 2670 | 20° 3’ 43.77’’ | 98° 31’ 20.90’’ |

**Table S3. Generalized linear models (GLMS) for the standardized effect sizes of functional richness (SES.FRic), functional evenness (SES.FEve), and functional divergence (SES.FDiv) of dung beetle assemblages in response to environmental variables. We show the three best models, and mark in bold the one with the lowest AIC value, which was considered as the best-fitted model.**

| Response variable | Models | Null Deviance | Residual Deviance | AIC |
| --- | --- | --- | --- | --- |
| **All sites** | | | | |
| **Functional richness** | **SES.FRic~Dung availability+Elevation** | **42.93** | **4.46** | **66.72** |
|  | SES.FRic~Elevation+Dung availability+Soil moisture | 42.93 | 24.29 | 68.59 |
|  | SES.FRic~Elevation+Dung availability+Land use heterogeneity | 42.93 | 24.37 | 68.65 |
| **Functional evenness** | **SES.FEve~Land use heterogeneity** | **12.17** | **11.25** | **49.97** |
|  | SES.FEve~Soil moisture | 12.17 | 11.99 | 51.17 |
|  | SES.FEve~Elevation | 12.17 | 12.01 | 51.20 |
| **Functional divergence** | **SES.FDiv~Soil moisture** | **16.52** | **12.29** | **51.64** |
|  | SES.FDiv~Soil moisture+Elevation | 16.52 | 11.31 | 52.06 |
|  | SES.FDiv~Soil moisture+Dung availability | 16.52 | 12.13 | 53.39 |
| **Forest sites** | | | | |
| **Functional richness** | **SES.FRic~Elevation+Dung availability+Soil hardness+Soil moisture+Land use heterogeneity+Percentage of pine-oak forest+Percentage of grassland** | **17.96** | **0.096** | **2.71** |
|  | SES.FRic~Elevation+Dung availability+Soil hardness+Soil moisture+Percentage of grassland | 17.96 | 0.191 | 4.870 |
|  | SES.FRic~Elevation+Dung availability+Soil hardness+Soil moisture+Percentage of pine-oak forest+Percentage of grassland | 17.96 | 0.172 | 5.904 |
| **Functional evenness** | **SES.FEve~Percentage of pine-oak forest** | **4.082** | **2.846** | **21.18** |
|  | SES.FEve~Elevation+Percentage of pine-oak forest | 4.082 | 2.774 | 22.95 |
|  | SES.FEve~Soil hardness+Percentage of pine-oak forest | 4.082 | 2.750 | 22.87 |
| **Functional divergence** | **SES.FDiv~Elevation+Dung availability+Soil hardness+Soil moisture+Percentage of pine-oak forest+Percentage of grassland** | **6.197** | **0.885** | **20.67** |
|  | SES.FDiv~Elevation+Dung availability+Soil hardness+Soil moisture+Land use heterogeneity+Percentage of pine-oak forest+Percentage of grassland | 6.197 | 0.803 | 21.79 |
|  | SES.FDiv~Elevation+Dung availability+Soil hardness+Land use heterogeneity+Percentage of pine-oak forest+Percentage of grassland | 6.197 | 1.144 | 22.97 |
| **Grassland sites** | | | | |
| **Functional richness** | **SES.FRic~Elevation** | **17.01** | **11.05** | **35.38** |
|  | SES.FRic~Elevation+Percentage of pine-oak forest | 17.01 | 10.70 | 37.05 |
|  | SES.FRic~Elevation+Percentage of grassland | 17.01 | 10.86 | 37.20 |
| **Functional evenness** | **SES.FEve~Percentage of grassland** | **7.563** | **5.377** | **28.18** |
|  | SES.FEve~Dung availability+Percentage of grassland | 7.563 | 4.582 | 28.57 |
|  | SES.FEve~Soil moisture+Percentage of grassland | 7.563 | 4.969 | 29.39 |
| **Functional divergence** | **SES.FDiv~Elevation+Dung availability+Soil moisture** | **7.314** | **0.798** | **13.10** |
|  | SES.FDiv~Dung availability+Percentage of grassland | 7.314 | 1.008 | 13.43 |
|  | SES.FDiv~Dung availability+Soil moisture | 7.314 | 1.060 | 13.94 |

**Supplementary Method: Functional traits of dung beetle species**

For the food relocation behaviour there are three categories: dwellers, tunnellers and rollers. Dwellers are those species that place their nests inside the food source; tunnellers dig tunnels and galleries under the soil to later bury part of the dung below the place it was deposited, and rollers make balls of manure as a source of food and oviposition, roll and bury it far from the source. For the activity period, there are two categories: diurnal and nocturnal beetles. Likewise, for habit diet preference: coprophagous or copro-necrophagous. The coprophagous species are those that preferentially feed on manure, while the copro-necrophagous are species attracted to manure, but that also feed on carrion. All these functional traits were obtained from the literature^2–13^. We selected these functional traits because they contribute to describe the role of dung beetles within ecosystems^3,9,14^. The food relocation behaviour and size are related to the amount and type of decomposing material that can be buried by the species^2,15,16,17^; as well as to their ability to colonize the resource and to coexist with potential competitors^16^. The activity period is related to the temporal opportunity to process decomposing materials^17^, while diet preference is determined by their nutritional requirements^17^, anatomical and physiological adaptations. Overall, food relocation behaviour, activity period and diet depend on the nesting requirements and metabolism of the species^2,16,18^, and reflect the role of these beetle species in dung removal and relocation, therefore being critical for ecosystem functions such as bioturbation, nutrient cycling and regulation of greenhouse gases emission.

Morphological traits are also assumed to reflect how dung beetles take advantage of the ecological resources due to their body shape and size^11,19^, and they promote the sensitivity of the components of functional diversity^19^. Thus, we included the average of the following morphological measurements: 1) body length, 2) body width, 3) dorso-ventral length, 4) clypeus length, 5) head length, 6) pronotum length, 7) abdomen length, 8) forelegs (protibia and protarsus) length and 9) hindlegs (metatibia and metatarsus) length. These morphological traits are expected to be related with dispersal capacity, competitive strength, and food manipulation. Besides that, body length, legs length and abdomen size of dung beetles predict some behavioural traits, body size and reproductive capacity^20^. Moreover, body width, dorso-ventral length, head length, pronotum length and forelegs and hindlegs length are closely associated with speed, force and pushing capacity (vertical or horizontal) of soil and decomposing material. Thus, the effectiveness of dung removal and relocation depends on the pressure exerted by the frontal area of the beetle^21,22^. In addition, these morphological traits could reflect differences in the ability of movement in different types of soils^22,23^.

The capacity of flight is another important functional trait because it is usually related to the reproductive success of the species, its facility to arrive to the food source when it is fresh, and this trait is influenced by the environmental temperature. Consequently, we included wing loading as a functional trait, and it was calculated as the division of body mass (mg) per the wing surface (mm^2^)^24^. A low wing loading facilitates the species’ flight and the location of the food source^9,24^. We measured the total wing surface as the sum of the wing area of each pair of wings. For this, we removed the pair of wings from the body and put them on a slide with a rapid mounting fixative solution (xylene). Then, we took a photograph of the wings with a 16 megapixel Nikon Coolpix Aw120 digital camera. The wing area was obtained with the ImageJ digital image processing program (version 1.51j8, developed by the National Institute of Health, USA).

Finally, biomass (dry weight) was included to determine the importance of the functionality of each species, because it is strongly related with the metabolism of the species^25,26^ and therefore with the amount of energy and resources assimilated^25,27^. For this, the dry weight of the collected specimens was obtained, placing them in an oven at 50ºC for 48 h. Then, they were weighed with an ultra-micro analytical balance (Thermo Cahn C-35, 10 µg precision). The values obtained for all the functional traits are shown in Table S4.

To obtain the morphological traits, wing loading and biomass, we used 20 randomly selected individuals of each species; for species with sexual dimorphism we measured 10 males and 10 females. For 10 species that were recorded with lower numbers of captured beetles, we measured all the available individuals (from 1 to 20). Given this low abundance, it was not possible for us to measure individuals from each habitat type or site. However, further studies may be focused on detecting intraspecific differences in traits between both types of habitats in order to assess habitat filtering at the individual level.

An important issue to take into account is that the procedure that we used to calculate functional diversity indices corrects the redundancy in the selected traits. Many traits, especially the morphological ones, are correlated. Thus, before computing the indices, the traits distance matrix is analyzed through a Principal Coordinates Analysis (PCoA) to represent species distribution in a multidimensional functional space, i.e., the coordinates of species in a Euclidean functional space with reduced uncorrelated dimensions. The resulting PCoA axes are used as the new traits to compute the functional diversity indices, avoiding redundancy and standardizing values.

**Table S4. Functional traits of dung beetle species (Scarabaeoidea) collected, and their total abundance (number of individuals) in forest and grassland sites.**

| **Species** | **Biomass (mg)** | **WL (mg/mm2)** | **BL (mm)** | **BW (mm)** | **DVL (mm)** | **CL(mm)** | **HL (mm)** | **PL (mm)** | **AL (mm)** | **FL (mm)** | **FL (mm)** | **HL (mm)** | **HL (mm)** | **FR** | **AP** | **Diet type** | **Forest*** | **Grassland*** |
| --- | --- | --- | --- | --- | --- | --- | --- | --- | --- | --- | --- | --- | --- | --- | --- | --- | --- | --- |
| **Scarabaeidae, Aphodiinae** | | | | | | | | | | | | | | | | | | |
| *Agrilinellus antonioreyi* Dellacasa, Dellacasa & Gordon, 2008 | 0.57 | 0.12 | 3.77 | 1.55 | 1.45 | 0.20 | 0.30 | 0.82 | 2.09 | 0.98 | 0.98 | 1.77 | 1.77 | D | N | C | 0 | 3 |
| *Agrilinellus ornatus* (Schmidt, 1911) | 0.98 | 0.10 | 3.71 | 1.36 | 1.04 | 0.17 | 0.47 | 0.68 | 1.91 | 0.89 | 0.89 | 1.15 | 1.15 | D | N | C | 3 | 0 |
| *Ataenius heinekeni* (Wollaston, 1854) | 1.17 | 0.08 | 4.54 | 1.46 | 1.30 | 0.23 | 0.58 | 0.94 | 2.65 | 0.73 | 0.72 | 1.39 | 1.39 | D | N | C | 4 | 32 |
| *Cephalocyclus fuliginosus* (Harold, 1863) | 2.14 | 0.04 | 6.48 | 2.28 | 1.97 | 0.24 | 0.61 | 1.14 | 4.23 | 1.48 | 1.42 | 2.26 | 2.11 | D | N | C | 7 | 0 |
| *Cephalocyclus mexicanus* (Harold, 1862) | 2.19 | 0.05 | 5.98 | 2.24 | 1.86 | 0.21 | 0.59 | 1.04 | 3.60 | 1.42 | 1.39 | 2.67 | 2.67 | D | N | C | 3 | 12 |
| *Gonaphodiellus opisthius* (Bates, 1887) | 0.99 | 0.05 | 4.20 | 1.68 | 1.28 | 0.20 | 0.47 | 0.70 | 2.49 | 0.72 | 4.02 | 1.37 | 1.38 | D | N | C | 570 | 487 |
| *Labarrus cincticulus* Hope, 1847 | 1.73 | 0.10 | 5.18 | 1.94 | 1.56 | 0.23 | 0.59 | 0.90 | 2.82 | 0.93 | 0.92 | 1.49 | 1.49 | D | N | C | 0 | 44 |
| *Labarrus pseudolividus* (Balthasar, 1941) | 1.59 | 0.07 | 5.02 | 2.00 | 1.63 | 0.21 | 0.48 | 1.21 | 2.81 | 1.03 | 0.80 | 1.75 | 1.73 | D | N | C-N | 0 | 6 |
| *Oscarinus indutilis* (Harold, 1874) | 1.48 | 0.06 | 4.80 | 1.90 | 1.65 | 0.23 | 0.55 | 0.77 | 2.49 | 1.08 | 1.12 | 1.47 | 1.46 | D | N | C | 4 | 36 |
| *Planolinellus vittatus* (Say, 1825) | 1.34 | 0.32 | 4.06 | 1.67 | 1.96 | 0.27 | 0.53 | 1.12 | 2.04 | 0.71 | 0.71 | 1.43 | 1.43 | D | N | C-N | 0 | 4 |
| *Pseudagolius coloradensis* (Horn, 1870) | 3.41 | 0.08 | 6.47 | 2.61 | 2.29 | 0.32 | 0.73 | 1.10 | 3.70 | 1.30 | 1.28 | 2.12 | 2.00 | D | N | C | 0 | 34 |
| **Geotrupidae, Geotrupinae** | | | | | | | | | | | | | | | | |  |  |
| *Ceratotrupes bolivari* Halffter & Martinez, 1962 | 132.48 | 0.79 | 18.32 | 10.13 | 7.49 | 1.27 | 3.89 | 5.02 | 10.10 | 7.19 | 7.19 | 8.09 | 8.09 | T | N | C | 3 | 0 |
| *Geotrupes nebularum* (Howden, 1864) | 143.12 | 0.8 | 20.59 | 10.39 | 7.82 | 1.11 | 3.66 | 5.95 | 11.18 | 7.61 | 7.61 | 9.37 | 9.37 | T | N | C-N | 1 | 0 |
| ***Scarabaeidae, Scarabaeinae*** | | | | | | | | | | | | | | | | | | |
| *Canthon (Canthon) humectus hidalgoensis* Bates, 1887 | 56.47 | 0.55 | 14.72 | 7.67 | 5.99 | 1.44 | 3.41 | 4.14 | 7.22 | 3.95 | 3.96 | 6.74 | 6.02 | R | D | C-N | 14 | 64 |
| *Copris armatus* Harold, 1869 | 171.91 | 0.61 | 20.70 | 10.36 | 8.25 | 1.41 | 4.09 | 6.21 | 10.23 | 4.96 | 5.00 | 7.61 | 7.42 | T | N | C-N | 48 | 91 |
| *Euoniticellus intermedius* (Reiche, 1848) | 9.41 | 0.14 | 8.95 | 3.47 | 2.84 | 0.46 | 1.16 | 3.19 | 3.73 | 2.05 | 1.99 | 3.00 | 3.00 | T | D | C-N | 0 | 2 |
| *Onthophagus chevrolati* Harold, 1869 | 15.52 | 0.33 | 8.54 | 4.75 | 3.17 | 0.66 | 1.87 | 2.78 | 3.69 | 2.29 | 2.29 | 3.41 | 3.24 | T | D | C | 150 | 191 |
| *Onthophagus gibsoni* Howden & Génier 2004 | 3.15 | 0.19 | 5.98 | 2.90 | 2.14 | 0.40 | 1.21 | 1.81 | 2.34 | 2.05 | 2.05 | 2.42 | 2.42 | T | D | C | 0 | 5 |
| *Onthophagus lecontei* Harold, 1871 | 4.29 | 0.25 | 6.02 | 3.20 | 2.21 | 0.42 | 1.22 | 2.31 | 2.35 | 1.69 | 1.69 | 2.43 | 2.33 | T | D | C-N | 3 | 77 |
| *Onthophagus mexicanus* Bates, 1887 | 8.41 | 0.31 | 7.53 | 4.20 | 3.15 | 0.56 | 1.59 | 3.06 | 2.93 | 1.81 | 1.75 | 2.33 | 2.22 | T | D | C-N | 231 | 1313 |
| *Phanaeus adonis* Harold, 1863 | 114.38 | 0.72 | 17.00 | 9.59 | 7.00 | 1.72 | 4.20 | 5.38 | 7.32 | 4.12 | 4.14 | 6.75 | 7.03 | T | D | C-N | 0 | 5 |
| *Phanaeus palliatus* Sturm, 1843 | 136.04 | 0.92 | 17.76 | 10.87 | 7.62 | 2.26 | 3.70 | 6.75 | 7.72 | 4.47 | 5.43 | 7.48 | 7.48 | T | D | C | 8 | 131 |
| *Phanaeus quadridens* (Say, 1835) | 179.27 | 1.07 | 19.35 | 11.48 | 8.41 | 2.17 | 4.43 | 6.85 | 8.20 | 5.56 | 5.54 | 8.46 | 8.47 | T | D | C-N | 0 | 53 |

BL-body length, BW-body width, DVL-dorso-ventral length, CL-clypeus length, HL-head length, PL-pronotum length, AL-abdomen length, FL-forelegs (protibia and protarsus) length and HL-hindlegs (metatibia and metatarsus) length. FR-food relocation (R-rollers, T-tunnellers, D-dwellers), AP-activity period (D-diurnal, N-nocturnal), diet type (C-coprophagous, C-N- copro-necrophagous). *Abundance.

**Supplementary Method: Method used to characterize the heterogeneity of land uses surrounding the sampling sites**

In each sampling site, an aerial image was taken with a drone (Phantom 3 advanced model, 12-megapixel camera, GPS + GLONASS) located at the centre of the set of traps. The images obtained have a resolution of 0.16 meters and were georeferenced using Google Earth images. Using these images, and field information, the following land uses (coverage types) were identified (Table S5): 1) pine-oak forest, 2) crops (corn, oats or wheat), 3) human constructions (roads), 4) grasslands, 5) bare ground, and 6) other types of non-forest vegetation (i.e., live fences, isolated trees, *Agave* plants, bushes).

To quantify the proportion of the area covered by each land use in the 19 sites, we used ArcGis (version 10.2, ESRI Inc., Redlands, CA, USA) with the following method: 1) the location of the traps was added to the photographs; 2) a minimum convex polygon was drawn using the location of the traps as vertices; 3) with the geoprocessing tool, dissolved buffers of 100 m were created from the vertices of the polygons or polylines according to each case; 4) we did a supervised digitization of the different land-use types within the buffers corroborated satellite images of Google Earth with the information collected in the field; and 5) the areas of the digitized polygons for each land-use type were quantified and their percentages from the buffer area were calculated. Lastly, the spatial heterogeneity of each sampling site was measured as the diversity of land uses in buffers, using the exponential of the Shannon-Wiener entropy index (^1^D), *sensu* Jost^28^, i.e. the effective number of land-use types. We considered as most heterogeneous the sites with the highest values of land use diversity^29^.

**Table S5. Percentages of each land use type in buffers of 100 m around the pitfall traps used for capturing dung beetles.**

| **Site** | **Pine-oak forest** | **Grassland** | **Human constructions** | **Crops** | **Bare ground** | **Other types of non-forest vegetation** |
| --- | --- | --- | --- | --- | --- | --- |
| 1G | 18.47 | 22.36 | 4.51 | 24.26 | 4.15 | 26.41 |
| 2F | 96.94 | 0 | 0 | 0 | 3.07 | 0 |
| 3G | 97.20 | 0 | 0 | 0 | 2.86 | 0 |
| 4F | 66.77 | 25.15 | 3.16 | 0 | 2 | 1.45 |
| 5G | 14.53 | 71.7 | 0 | 5.38 | 0.92 | 7.64 |
| 6F | 80.01 | 0 | 0 | 2.42 | 17.62 | 0 |
| 7G | 34.60 | 39.45 | 0.30 | 10.97 | 7.15 | 7.67 |
| 8F | 97.64 | 0 | 0.25 | 0 | 2.12 | 0 |
| 9G | 22.41 | 39.02 | 0.20 | 24.81 | 0 | 13.67 |
| 10F | 100 | 0 | 0 | 0 | 0 | 0 |
| 11G | 1.88 | 71 | 2.62 | 3.16 | 2.97 | 18.58 |
| 12F | 89.63 | 6.41 | 0.4 | 0.22 | 1.44 | 2.01 |
| 13G | 32.44 | 34.72 | 0 | 23.83 | 3.78 | 5.49 |
| 14F | 84.55 | 0 | 0.64 | 10.79 | 3.22 | 0.9 |
| 15G | 4 | 69.48 | 0 | 0 | 0 | 26.63 |
| 16F | 99.80 | 0 | 0 | 0 | 0.25 | 0 |
| 17G | 91.99 | 7.07 | 0.55 | 0 | 0.49 | 0 |
| 18F | 52.76 | 39.20 | 0 | 0 | 5.29 | 2.88 |
| 19G | 65.43 | 32.78 | 0 | 0 | 1.88 | 0 |
| 20F | 99.05 | 0 | 0 | 0 | 0.95 | 0 |

**References**

1. Pavón, N. P. & Meza, S. M. Cambio climático en el estado de Hidalgo: clasificación y tendencias climáticas. (Universidad Autónoma del Estado de Hidalgo, 2009).
2. Halffter, G. & Edmonds, W. D. The nesting behavior of dung beetles (Scarabaeinae). An ecological and evolutive approach. (Instituto de Ecología, 1982).
3. Hanski, I. & Cambefort, Y. Dung beetle ecology. (Princeton University Press, 1991).
4. Terrón, R. A., Anduaga, S. & Morón, M. A. Análisis de la Coleopterofauna necrófila de la reserva de la biosfera "La Michilia”, Durango, México. *Folia Entomol. Mex.* **81**, 315–324, (1991).
5. Montes de Oca, E. Escarabajos coprófagos de un escenario ganadero típico de la región de los Tuxtlas, Veracruz, México: importancia del paisaje en la composición de un gremio funcional. *Acta Zool. Mex.* (82), 111–132, (2001).
6. Morón, M. A. Atlas de los escarabajos de México. Coleoptera: Lamellicornia Vol. II Familias Scarabaeidae, Trogidae, Passalidae y Lucanidae. (Argania Edition, S.C.P., 2003).
7. Verdú, J. R., Arellano, L., Numa, C. & Micó, E. Roles of endothermy in niche differentiation for ball‐rolling dung beetles (Coleoptera: Scarabaeidae) along an altitudinal gradient. *Ecol. Entomol.* **32**(5), 544–551, https://doi.org/10.1111/j.1365-2311.2007.00907.x (2007).
8. Price, D. L. & May, M. L. Behavioral ecology of *Phanaeus* dung beetles (Coleoptera: Scarabaeidae): review and new observations. *Acta Zool. Mex. Nueva Ser.* **25**, 211–238, https://doi.org/10.21829/azm.2009.251621 (2009).
9. Scholtz, C. H., Davis, A. L. V. & Kryger, U. Evolutionary biology and conservation of dung beetles. (Pensoft, 2009).
10. Barragán, F., Moreno, C. E., Escobar, F., Halffter, G. & Navarrete, D. Negative impacts of human land use on dung beetle functional diversity. *PLoS ONE* **6**(3), e17976, https://doi.org/10.1371/journal.pone.0017976 (2011).
11. Simmons, L. W. & Ridsdill-Smith, T. J. Ecology and evolution of dung beetles. (John Wiley & Sons, 2011).
12. Pérez-Villamares, J. C., Jiménez-Sánchez, E. & Padilla-Ramírez, J. Escarabajos atraídos a la carroña (Coleoptera: Scarabaeidae, Geotrupidae, Hybosoridae, Trogidae y Silphidae) en las cañadas de Coatepec Harinas, Estado de México, México. *Rev. Mex. Biodivers.* **87**(2), 443–450, https://doi.org/[10.1016/j.rmb.2016.03.005](https://doi.org/10.1016/j.rmb.2016.03.005) (2016).
13. Trujillo-Miranda, A. L., Carrillo-Ruiz, H., Rivas-Arancibia, S. P. & Andrés-Hernández, A. R. Estructura y composición de la comunidad de escarabajos (Coleoptera: Scarabaeoidea) en el cerro Chacateca, Zapotitlán, Puebla, México. *Rev. Mex. Biodivers.* **87**(1), 109–122, https://doi.org/10.1016/j.rmb.2015.08.008. (2016).
14. Nichols, E. *et al.* Ecological functions and ecosystem services provided by Scarabaeinae dung beetles. *Biol. Cons.* **141**(6), 1461–1474, https://doi.org/10.1016/j.biocon.2008.04.011 (2008).
15. Doube, B. M. A functional classification for analysis of the structure of dung beetle assemblages. *Ecol. Entomol.* **15**(4), 371–383, https://doi.org/10.1111/j.1365-2311.1990.tb00820.x (1990).
16. Horgan, F. G. Burial of bovine dung by coprophagous beetles (Coleoptera: Scarabaeidae) from horse and cow grazing sites in El Salvador. *Eur. J. Soil Biol.***37**(2), 103–111, https://doi.org/10.1016/S1164-5563(01)01073-1 (2001).
17. Horgan, F. G. Dung beetle assemblages in forests and pastures of El Salvador: a functional comparison. *Biodivers. Conserv.* **17**(12), 2961, https://doi.org/10.1007/s10531-008-9408-2 (2008).
18. Doube, B. M., Giller, P. S. & CSIRO, F. M. Dung burial strategies in some South African coprine and onitine dung beetles (Scarabaeidae: Scarabaeinae). *Ecol. Entomol.* **13**(3), 251–261, https://doi.org/10.1111/j.1365-2311.1988.tb00354.x (1988).
19. Voß, K. & Schäfer, R. B. Taxonomic and functional diversity of stream invertebrates along an environmental stress gradient. *Ecol. Indic.* **81**, 235–242. https://doi.org/10.1016/j.ecolind.2017.05.072 (2017).
20. Raine, E. H., Gray, C. L., Mann, D. J. & Slade, E. M. Tropical dung beetle morphological traits predict functional traits and show intraspecific differences across land uses. *Ecol. Evol.* **8**(17), 8686–8696, https://doi.org/10.1002/ece3.4218 (2018).
21. Forsythe, T. G. Running and pushing in relationship to hind leg structure in some Carabidae (Coleoptera). *Coleopt. Bull.* **35**(4), 353–378, https://doi.org/10.2307/4007954 (1981).
22. Evans, M. E. G. & Forsythe, T. G. A comparison of adaptations to running, pushing and burrowing in some adult Coleoptera: especially Carabidae. *J. Zool.* **202**(4), 513–534. doi:10.1111/j.1469-7998.1984.tb05049.x (1984).
23. Forsythe, T. G. Locomotion in ground beetles (Coleoptera carabidae): An interpretation of leg structure in functional terms. *J. Zool.* **200**(4), 493–507, https://doi.org/10.1111/j.1469-7998.1983.tb02811.x (1983).
24. Pennycuick, C. J. Bird flight performance: a practical calculation manual. (UniversityPress, 1989).
25. Saint-Germain, M. *et al.* Should biomass be considered more frequently as a currency in terrestrial arthropod community analyses? *J. Appl. Ecol.* **44**, 330–339, https://doi.org/10.1111/j.1365-2664.2006.01269.x (2007).
26. Shahabuddin, *et al.* Diversity and body size of dung beetles attracted to different dung types along a tropical land-use gradient in Sulawesi, Indonesia. *J. Trop. Ecol.* **26**, 53–65, https://doi.org/10.1017/S0266467409990423 (2010).
27. Brown, J. H., Gillooly, J. F., Allen, A. P., Savage, V. M. & West, G. B. (2004). Toward a metabolic theory of ecology. *Ecology* **85**(7), 1771–1789, https://doi.org/10.1890/03-9000 (2004).
28. Jost, L. Entropy and diversity. *Oikos* **113**(2), 363–375. https://doi.org/10.1111/j.2006.0030-1299.14714.x (2006).
29. Fahrig, L. *et al.* Functional landscape heterogeneity and animal biodiversity in agricultural landscapes. *Ecol. Lett.* **14**(2), 101–112. https://doi.org/10.1111/j.1461-0248.2010.01559.x (2011).
